# Calprotectin: An Ignored Biomarker of Neutrophilia in Pediatric Respiratory Diseases

**DOI:** 10.3390/children8060428

**Published:** 2021-05-21

**Authors:** Grigorios Chatziparasidis, Ahmad Kantar

**Affiliations:** 1 Primary Cilia Dyskinesia Unit, School of Medicine, University of Thessaly, 41110 Thessaly, Greece; gchatziparasidis@gmail.com; 2 Pediatric Asthma and Cough Centre, Instituti Ospedalieri Bergamaschi, University and Research Hospitals, 24046 Bergamo, Italy

**Keywords:** calprotectin, S100A8/A9, children, lung

## Abstract

Calprotectin (CP) is a non-covalent heterodimer formed by the subunits S100A8 (A8) and S100A9 (A9). When neutrophils become activated, undergo disruption, or die, this abundant cytosolic neutrophil protein is released. By fervently chelating trace metal ions that are essential for bacterial development, CP plays an important role in human innate immunity. It also serves as an alarmin by controlling the inflammatory response after it is released. Extracellular concentrations of CP increase in response to infection and inflammation, and are used as a biomarker of neutrophil activation in a variety of inflammatory diseases. Although it has been almost 40 years since CP was discovered, its use in daily pediatric practice is still limited. Current evidence suggests that CP could be used as a biomarker in a variety of pediatric respiratory diseases, and could become a valuable key factor in promoting diagnostic and therapeutic capacity. The aim of this study is to re-introduce CP to the medical community and to emphasize its potential role with the hope of integrating it as a useful adjunct, in the practice of pediatric respiratory medicine.

## 1. Introduction 

In 1965, Moore published an article about newly discovered proteins found in the nervous system of the bovine brain. They were partly soluble in 100% saturated ammonium sulfate, so they were named S100 proteins [1]. They belong to a Ca^2+^-binding superfamily that comprises many proteins, including S100A8 and S100A9 [2]. About three decades later, Rammes et al. coined the terms myeloid-related proteins 8 and 14 (MRP8 and MRP14, respectively) in light of the primary expression of S100A8 and S100A9 in myeloid lineage cells [3]. Later, the nomenclature was refined, and the proteins were renamed calgranulin A and calgranulin B based on their Ca^2+^-binding properties [4]. 

The term calprotectin (CP) was coined only after its role in inflammatory processes in the human body was discovered (the terms S100 proteins, S100A8/A9, MRP8/MRP14, calgranulin A and B, which are all synonyms of calprotectin, are still used by some authors). CP was identified as a promising marker of inflammation or nutrient antagonism occurring within the organism for the first time in 1984 [5]. Numerous reports since then have emphasized the critical role of CP in human defense mechanisms, and it has been extensively studied as a surrogate marker of inflammatory bowel disease. At the same time, however, it is only beginning to be understood in pediatric respiratory care.

## 2. CP Structure and Genes

CP is a 24-kD heterodimer that is a member of the S100 protein family. It shares a helix-loop-helix motif structure with S100 proteins, consisting of two helices connected by a central hinge region [6]. Each monomer contains two EF-hand motifs that can be coupled to two Ca^2+^ ions or other divalent metal ions such as zinc (Zn^2+^). The binding capacity of S100 proteins to Ca^2+^ has no effect on the zinc-binding capacity. Additionally, histidine-binding sites contribute to the antibacterial properties of CP [7].

The CPs role in ion binding significantly influences their activity. S100 proteins (S100A8/A9) that form CP translocate to the plasma membrane and intermediate filaments in a calcium-dependent manner. S100 proteins are able to affect the activity of other proteins by adjusting extracellular and intracellular concentrations of calcium, zinc, and copper. Binding to metal ions initiates oligomerization and the folding of S100 proteins, leading to their regulation [8].

As previously stated, each S100A8 and S100A9 monomer contains two EF-hand motifs with varying Ca^2+^ affinity. Ca^2+^ binding causes a conformational change that influences target recognition [9]. Additionally, the CP complex exhibits a high affinity for Zn^2+^ and Mn^2+^ binding. [10]. The affinity of CP for Zn^2+^ and Mn^2+^ could be a possible explanation for its antimicrobial properties, as CP sequesters these elements from the surroundings [11]. 

Vogl et al. [12] demonstrated that, while CP can form heterodimers spontaneously in the absence of metal ions, tetramers of S100A8/S100A9 are strictly dependent on the presence of Ca^2+^ or Zn^2+^. There is as yet no scientific consensus about the biological significance of intracellular S100A8 and S100A9 heterodimers. Nonetheless, heterotetramerization appears to be critical for CP intracellular functions, as failure to form tetramers is associated with a loss of within-cell function [13]. 

CP is encoded by genes located in the chromosomal region 1q21, which also encodes the vast majority of S100 proteins, except for S100B, S100P, and S100Z [14,15].

## 3. Mechanisms of CP Release

Passive and active mechanisms mediate the release of CP from myeloid cells into the extracellular space. Tissue damage, cellular necrosis [16], and neutrophil extracellular trap (NET) formation [17] can all cause the cytosolic protein complex to be released passively. The heterodimer must be transported from the cytosol to the plasma membrane in a Ca^2+^-dependent manner during active release [18]. However, at the cellular level, the classical secretion route is mediated by the Golgi apparatus, and the CP complex lacks the structural requirements for this. It is therefore reasonable to assume that an alternative secretory route controls the active secretion of CP.

Human leukocytes are capable of actively secreting CP in an energy-dependent manner via protein kinase-C and an intact microtubule network. However, neither the classical protein secretion route nor the interleukin-1 alternative protein secretion route [3] are used in the release process. It appears that C5a and formyl peptide (fMLP) stimulation regulate the release mechanism [19]. Frosch et al. reported that when monocytes interact with activated TNF-α, CP is released, implying that endothelial inflammatory changes can act as a trigger [20]. The endothelium is specifically adjusted to optimize leukocyte recruitment in the presence of inflammation. The leukocytes are recruited after a well-defined activation cascade [21] that starts with the engagement of endothelial expressed selectins. Leucocyte rolling along the endothelium is mediated by P-selectin glycoprotein ligand 1 (PSGL1), which enables endothelial chemokines to interact with the corresponding chemokine receptors on leucocytes. The β^2^ integrins expressed on leucocytes are subsequently subject to a conformational change to help cells adhere strongly [22].

Pruenster et al. demonstrated, in 2015, that the binding of PSGL1 to E-selectin results in rapid CP secretion by neutrophils in vitro and in vivo [23]. In a TNF-α-induced inflammation model, the same team discovered that neutrophils are the main source of serum CP. To corroborate this, neutrophil-depleted mice had significantly lower serum CP levels. Additionally, when they blocked E-selectin in vivo with a blocking antibody, they observed a dramatic decrease in the serum levels of CP in the same model, demonstrating the specificity of the E-selectin–PSGL1 interaction in the rapid release of CP from neutrophils (Table 1).

## 4. Distribution and Reference Range of CP in Humans

CP is predominantly found in neutrophils and a subpopulation of reactive tissue macrophages, with some expression in endothelial and epidermal cells [24]. It accounts for approximately half of the total cytosolic protein in neutrophils [25]. It is also found in non-keratinizing squamous epithelia, renal tubules, and some mucosal epithelial cells [26]. 

The free/soluble form of CP can be found in serum with a reference range of 1–6 mg/L in healthy people. In active inflammation, a 100-fold increase in serum CP levels may be observed [27]. Although gender does not have any influence on serum CP levels [28], it does not seem to apply in obese and overweight children, where levels are higher than in normal-weight subjects [29]. CP can also be found in urine, body secretions, intestinal fluid, and feces [30]. It is reported that the normal range of fecal CP lies between 10 and 50 mg/L [27].

However, fecal CP levels should be viewed with caution because children under the age of four have naturally higher levels [31] and an upward trend has been shown before the age of four years [32]. Children aged 2–9 years old have substantially higher fecal CP concentrations than subjects aged over 10 years old, according to other studies [33]. Fecal CP appears to increase with age in children with cystic fibrosis (CF), with levels exceeding 50 mg/kg in children aged 4 to 10 years. [31].

## 5. The Importance of Membrane-CP Interaction

To eliminate the inflammatory trigger and aid tissue repair, circulating blood leukocytes must migrate to sites of tissue injury and infection [34]. The development of weak and temporary adhesive interactions between the white cells and endothelial cells of postcapillary venular walls near inflamed tissues is the first step in leukocyte migration [21]. Heparin sulfate (HS), a linear polysaccharide present in all animal tissues, is involved in leukocyte attachment. [35].

HS is a proteoglycan located near extracellular matrix proteins and cell surfaces [36]. HS is found in endothelial cells, and in vitro studies have shown that it interacts with the MRP-14 subunit of CP, thus facilitating the extravasation of leukocytes [37,38,39,40]. After trans-endothelial migration, leukocytes release CP, thus increasing the CP concentration in the body fluids of patients with acute or chronic inflammatory conditions [41,42].

The role of CP in Th2 inflammation has received a lot of attention in recent years. Thymic stromal lymphopoietin (TSLP) and IL-25, two newly discovered epithelial-derived cytokines, can induce Th2–cytokine-dependent inflammation and play a role in innate and adaptive immune responses in the airway mucosa [43]. Allergic stimulants such as *Alternaria* *alternata* and house dust mites (HDM) cause the airway epithelial cells to produce TSLP and IL-25 through the protease-activated receptor (PAR)-2 [44]. In allergic rhinitis, asthma, and atopic dermatitis, these cytokines play a role in the initiation and progression of allergic inflammation [45]. In cultured normal human bronchial epithelial cells, airborne allergens like *Alternaria* and HDM stimulate the production and secretion of CP, where the combined effect of CP and ATP induces the production of TSLP and IL-25. These findings indicate that CP, through the secretion of TSLP and IL-25, promotes allergen-induced Th2-type inflammatory responses in airway epithelial cells, and that CP released by epithelial cells may be involved in the pathogenesis of allergic diseases [46].

The S100 protein, uric acid, ATP, and high-mobility group box 1 (HMGB1) protein are all examples of damage-associated molecular patterns (DAMPs). DAMPs, also known as alarmins, are intracellular molecules that participate in cellular functions under normal homeostasis, but are released outside the cell when tissue damage occurs [47]. DAMPs and allergic inflammation have recently been related, and very high levels of HMGB1 have been discovered in the sputum and nasal secretions of patients with asthma, nasal allergy, or chronic rhinosinusitis [48,49,50]. 

## 6. The Importance of Soluble CP

In humans, the main role of CP is to engage in and mediate the inflammatory response. Infection-induced inflammation is, in fact, one of the key causes of CP secretion. As bacteria invade the body, the cells involved in innate immunity, such as neutrophils, macrophages, and monocytes, express and secrete CP. This occurs to regulate inflammation through the release of cytokines, reactive oxygen species (ROS), and nitric oxide (NO) [51]. CP also exhibits broad-spectrum antimicrobial activity against a variety of bacteria after being released into the extracellular space by recruited phagocytes, or after cell necrosis. The CPs ability to bind and regulate the levels of key trace metals including Zn^2+^ and Mn^2+^, which are necessary for bacterial development, underpins the entire procedure. Nutritional immunity is a term used to describe this mechanism [52].

Both Zn^2+^ and Mn^2+^ binding sites in CP must be functional, since a mutation in either site impairs antimicrobial activity [53]. Furthermore, since the local levels of Zn^2+^ and Mn^2+^ may modulate the affinity between CP and its targets, the antimicrobial properties of CP may vary in different pathological conditions. Aside from its metal-chelating abilities, CP has been shown to improve human neutrophil phagocytosis in a Syk-, Erk1/2-, and PI3K/Akt-dependent manner, enhancing its antimicrobial activity against *Klebsiella* *pneumoniae* and *Escherichia coli* [54].

The absence of CP causes a substantial increase in bacterial load in the blood, liver, and spleen in a mouse model [55]. Thus, during the early stages of infection, CP suppresses pathogen growth at infectious sites, allowing time for phagocyte recruitment. CP then boosts the phagocytic activity of the recruited leukocytes, hastening pathogen clearance. CP is released from the undamaged macrophages through DDX21–TRIF signaling during *Influenza A* virus infection, resulting in an exaggerated inflammatory response and cell death [56]. The duration of fever before admission, in *typhoid* fever patients, is linearly correlated with an increase in CP levels in acute-phase plasma and feces [57]. CP expression increases gradually until the patient dies of more serious infections associated with septic shock [58]. CP participates in innate immunity and mediates the inflammatory response, as shown by the early expression of S100 proteins during infection-induced inflammation. CP plays an important role in defending the body against invading pathogens via multiple TLR4- or RAGE-mediated inflammatory pathways [59]. CP is also involved in cytosol tubulin polymerization and cytoskeleton rearrangement, both of which are essential for cell migration. Its capacity to recruit neutrophils during inflammation is explained in part by this.

A negative feedback regulatory mechanism regulates the expression and secretion of CP during infection-induced inflammation [60]. The main goal of this mechanism is to suppress the excessive expression of CP, which amplifies the inflammatory response and causes neutrophils and macrophages to release more cytokines. The knockout breaks the vicious cycle that could exacerbate the disorder.

The S100A8 heterodimer is a TLR4 ligand that is strongly induced in endotoxic shock during a Gram-negative bacteria infection. High levels of S100A8 and S100A9 have been shown to stimulate RAGE signaling and cause inflammatory damage in septic shock patients [61]. S100A9 does not appear to increase in patients with an infectious flare up of chronic obstructive pulmonary disease (COPD), despite the fact that increased expression of S100 proteins has been linked to disease exacerbation. In this case, the decrease in S100A9 may indicate weakened immunity, which may explain the infection-induced exacerbation. Patients with extreme COPD caused by other causes, on the other hand, have a high expression of S100A9, indicating an uncontrolled immune response. Thus, both defense capabilities and immune homeostasis appear to require proper levels of S100 proteins [62].

## 7. CP and Respiratory Infections

Acute respiratory infections (ARIs) are the leading cause of death and morbidity across all age groups and genders in low- and middle-income countries (LMICs) [63,64]. ARIs are viruses or bacteria-related infections that are classified as lower respiratory tract infections (LRTIs) or upper respiratory tract infections (URTIs). The vast majority (97%) of acute lower respiratory infection (ALRI) cases occur in LMICs [65], where 6.9 million children died in 2011, with ALRI accounting for nearly one in five of these deaths [66]. In 2015, over 650,000 children under the age of five died as a result of LRTIs [67].

Although the underlying pathogenetic agents of acute respiratory infections vary considerably across the globe [68], early diagnosis is critical. There are two primary reasons for the early detection of ARIs. The first is to reduce the time interval between the onset of an ARI and the initiation of pathogen-specific treatment in order to decrease the risk of prolonged infection, sepsis, late respiratory sequelae, and even death [69]. Second, antibiotic resistance is a significant and growing global problem at the moment.

The initial enthusiasm following penicillin’s discovery in the first half of the 20th century was followed by concerns regarding emerging resistance, which was observed only a few years after the (over)application of penicillin in clinical medicine. Currently, judicious use of antibiotics remains of paramount importance, and despite the existence of clinical guidelines about the proper use of antibiotics in childhood infections, the medical community runs in circles [70]. Inappropriate, unnecessary, or overuse of the existing antibiotics has led to the re-occurrence of bacterial infections and has posed a threat to people’s health. It is obvious that practicing physicians must not delay diagnosis and treatment of ARIs, by they also have an obligation to avoid unselective antibiotic use.

It would be ideal if we could isolate and correctly identify the causative agent of an ARI promptly. Unfortunately, this is time consuming and not available in any setting. Therefore, we have to rely on blood tests for biomarkers to differentiate bacterial diseases from viral respiratory diseases. While the search for the ideal biomarker continues, there are currently no biomarkers capable of diagnosing bacterial ARI on their own [71]. A biomarker has been defined as “a characteristic that is objectively measured and evaluated as an indicator of normal biological processes, pathogenic processes, or pharmacologic responses to a therapeutic intervention” [72]. The ideal biomarker for bacterial ARIs should be elevated only when a bacterium causes the infection, not a virus or fungus, to underline the need of antibiotic treatment. Additionally, the ideal biomarker is expected to be inexpensive, simple to test, and informative within a short period of time [73].

There are numerous biomarkers available for clinical use, but each has inherent limitations. The white blood cell (WBC) count, erythrocyte sedimentation rate (ESR), C-reactive protein (CRP), and procalcitonin (PCT) are the most frequently used. The WBC count and ESR should not be used as biomarkers because they have a lower sensitivity and specificity than CRP and PCT [74]. A recent study examined whether serum CRP can be used to differentiate bacterial from viral pneumonia in 193 pediatric patients. CRP concentrations were found to have no significant correlation with the microbial etiology of pneumonia [75].

Furthermore, although PCT is considered more specific to identify a bacterial infection than other biomarkers, there are limitations to its use. The main one is the high rate of false-positive and false-negative results. For example, false-positive PCT values may occur in some viral infections, acute respiratory distress syndrome, inflammation associated with cytokine storm or cardiogenic shock. Furthermore, PCT measurement early in the course of an illness or in localized infections can give false-negatives [76].

Recently, the medical community’s desperate search for an ideal biomarker has shifted its focus to CP. The reason for this is that both bacterial and viral infections initiate an acute-phase response aimed at combating the infection and mitigating its effects. The innate immune system is activated first and most rapidly during infection, recruits other immune cells to the infection site, and activates both the complement cascade and WBCs to kill the microorganisms. The neutrophils are the first cells to reach the “battlefield” during this rapid process, and upon activation, they rapidly release neutrophil activation markers stored in the granulae or the cytoplasm [77]. Because no de novo synthesis is required, these markers may serve as an earlier indicator of neutrophil activation, in contrast to the delayed formation of new WBCs or acute-phase proteins. Since CP is one of the most abundant proteins in the neutrophil cytosol and is released upon activation, it theoretically meets some of the criteria for an ideal biomarker.

Havelka et al. compared the diagnostic accuracy of CP with that of heparin-binding protein (HBP) and PCT in ARIs [78]. They attempted to differentiate between patients with viral respiratory infections and those with bacterial pneumonia, *mycoplasma* pneumonia, and *streptococcal* tonsillitis (*n* = 135) by analyzing these biomarkers. The findings were compared to those of 144 healthy controls. Although all three biomarkers were increased in response to bacterial and viral infections, CP outperformed the other two. CP was significantly increased in patients with bacterial infections when compared to patients with viral infections (bacterial pneumonia, *mycoplasma* pneumonia, and *streptococcal* tonsillitis). The PCT levels were significantly higher in bacterial pneumonia than in viral infections, but were not significantly higher in *streptococcal* tonsillitis or *mycoplasma* infections. HBP performed even worse and was unable to differentiate bacterial from viral infections.

Fang et al. investigated the correlations between the serum S100A8 heterodimer and the severity of community-acquired pneumonia (CAP) and inflammatory cytokines in adults [79]. Another objective was to establish S100A8 cutoff values for predictive power in CAP patients. The study’s major findings were as follows: (1) serum S100A8 heterodimer levels were increased in CAP patients upon admission; (2) serum S100A8 heterodimer levels were positively associated with CAP severity scores; and (3) S100A8 knockdown attenuated the inflammatory cytokines induced by *Streptococcus pneumoniae* infection in human lung epithelial cells. This suggests that the S100A8 heterodimer may play a role in the initiation and progression of CAP. They concluded that in the future, the S100A8 heterodimer could be used as an early serum diagnostic biomarker and possibly as a therapeutic target for CAP.

Immunity homeostasis is dependent on adequate levels of S100 proteins, as demonstrated in an animal study examining the role of CP in the host response during *Staphylococcus aureus* pneumonia infection [80]. MRP14-deficient mice (mice unable to form CP) were inoculated intra-nasally with wild-type *S. aureus*. In the BAL and lung tissue, *S. aureus* pneumonia was associated with a significant increase in CP. Surprisingly, MRP14 deficiency had a negligible effect on *S. aureus* clearance, and was associated with increased cytokine levels in BAL and aggravated lung histopathology. It was also associated with decreased neutrophil transmigration into BAL at late time points following infection, as well as decreased nucleosome release. The study concluded that CP has an unexpected protective effect on the lungs during *staphylococcal* pneumonia.

A few years earlier, the same research team used the same model to determine the role of CP in *Klebsiella* pneumonia sepsis that originated from the lungs. They followed a well-established model in which bacteria grow gradually and then spread [55]. MRP14-deficient mice were unable to form MRP8/14 heterodimers, resulting in increased bacterial dissemination, organ damage, and decreased survival. MRP14-depleted macrophages exhibited decreased phagocytosis of *Klebsiella*. Furthermore, only recombinant MRP8/14 heterodimers, not MRP8 or MRP14 alone, inhibited *Klebsiella* growth in vitro via divalent cation chelation. *Klebsiella* growth was inhibited only by neutrophil extracellular traps (NETs) prepared from wild-type neutrophils, but not by neutrophils lacking MRP14. As a result of the study, MRP8/14 was identified as a critical component of protective innate immunity during *Klebsiella* pneumonia infection.

CP was evaluated as a biomarker for differentiating between bacterial and viral causes of CAP in hospitalized adults, as well as a potential predictor of post-discharge mortality. On admission, elevated CP levels were indicative of bacterial pneumonia. Surprisingly, 6-week CP levels were predictive of 5-year all-cause mortality [81].

Parapneumonic effusion (PPE) is defined as the accumulation of exudative fluids in the pleural cavity as a result of pneumonia [82]. It is challenging to identify the cause of PPE because patients experience the same clinical symptoms, such as a cough, chest pain and fever [83]. PPEs are classified as uncomplicated PPE (UPPE), complicated PPE (CPPE), or empyema based on the biochemical parameters of the pleural fluid, including pH, glucose, and lactate dehydrogenase (LDH) [84]. Currently, there is no single serum biomarker that can assist clinicians in distinguishing infectious from non-infectious PPE and guiding appropriate treatment [85].

Wu et al. sought to enhance the diagnostic power of these markers by combining them. They discovered that combining serum CRP and CP levels increased sensitivity to 73.52% and specificity to 80.55% for correctly diagnosing CPPE and empyema [86]. Notably, it was discovered that patients with malignant PPE had significantly lower pleural CP levels than those with infectious PPE. Sensitivity was 96.7% and specificity was 100% (*p* < 0.001) [87].

Serum CP levels were found to be elevated in patients with tuberculosis (TB), and when combined with other biomarkers, they may aid in diagnosing and treating this disease. Globally, tuberculosis is the second leading cause of infectious disease death. The primary impediment to global tuberculosis control continues to be early diagnosis. Xu et al. discovered statistical differences between tuberculosis (TB) and other lung disease cases in three serum proteins (S100A9, SOD3, and MMP9). The combination of these three biomarkers has the potential to differentiate tuberculosis from healthy controls [88]. Additionally, another study found that plasma a-1-acid glycoprotein (ORM2), IL-36A, and SOD1 exhibit 90% sensitivity and 89.66% predictive value in distinguishing between those with severe TB and mild TB, and healthy controls. Furthermore, ORM2, S100A9, IL-36α, and SOD1 were found to be positively correlated with the development of TB [89]. 

The concentration of CP was quantified in sputum and serum samples taken from patients with bronchiolitis obliterans (BO). Spirometric parameters were compared to the healthy controls. In sputum, CP levels were significantly elevated, which reflects ongoing neutrophilic inflammation and strongly correlates with FEV1 and MEF25. In comparison to the controls, serum CP levels were significantly lower in BO patients [90].

Table 2 and Table 3 depict a summary of human and animal studies focusing on the use of CP in infectious diseases. There is a paucity of pediatric studies that concern the implication of CP in diagnosis and the management of respiratory infections. More pediatric studies are needed if we are to incorporate CP in clinical practice.

### 7.1. CP in Cystic Fibrosis (CF) Patients

CP was first identified in CF lung secretions in 1975 [91,92], and was later dubbed CF antigen [92]. Gray et al. measured sputum and serum CP levels during and after a CF exacerbation and discovered that serum CP decreased four-fold following exacerbation treatment. This finding had a greater statistical significance than CP in sputum, implying that serum sampling is less variable than sputum sampling. Additionally, they discovered that a serum CP concentration <9.1 μg/mL at the end of an exacerbation predicted a longer time between exacerbations when compared to patients with CP > 9.1 μg/mL [93]. 

Additionally, elevated levels of CP were detected in the patient’s feces during a CF exacerbation. The finding was hypothetically attributed to the multiorgan dysbiosis that occurs in cystic fibrosis and was assumed to reflect a systemic exacerbation of the disease rather than a purely pulmonary manifestation. The exacerbation treatment decreased CP levels in the feces, possibly through an effect on the intestinal microbiome [94]. Serum CP levels in the stable state of cystic fibrosis can predict the time until the next exacerbation and the decline in lung function, allowing the treating physician to intervene earlier. Reid et al. [95] report that the rate of forced vital capacity decline accelerates by approximately 50 mL/year with a two-fold increase in baseline serum CP, and is independent of all the other variables tested. The available data indicate that CP may be a useful biomarker for predicting, diagnosing, and treating CF exacerbations.

### 7.2. CP in Non-CF Bronchiectasis (Non-CF BE)

The role of CP in the management of patients with non-CF BE has yet to be discovered. Only one study has looked for sputum biomarkers in patients with chronic suppurative lung disease [96]. The study reported the presence of calgranulin a, b, and c in abundance compared to healthy controls. There have been no reports on the levels of CP in protracted bacterial bronchitis. More studies are needed to clarify the role of CP in monitoring patients with non-CF BE since in the majority, the pathophysiology of the disease points to chronic neutrophilic inflammation.

### 7.3. CP in Asthmatics

The role of CP in the pathogenesis of asthma has attracted scientific interest. The S100A9 levels in sputum are significantly higher in patients with severe and neutrophilic-predominant asthma than in those with eosinophil-predominant and paucigranulocytic endotypes [97]. This could indicate that S100A9 initiates and exacerbates neutrophilic inflammation in these patients. However, the role of CP may vary depending on the type of underlying lung inflammation.

Palmer et al. used the *A. alternata* model of type 2 high allergic airway inflammation to conduct experiments on CP-deficient mice [98]. CP deficiency exacerbated airway eosinophilia, activated Th2 helpers, and increased methacholine-induced airway resistance and elastance. The authors hypothesize that the increased allergic inflammation was caused by T regulatory cells’ inability to control Th2 responses in the absence of CP. Lee et al. discovered that serum CP levels were correlated with lung function, airway hyperresponsiveness, and the percentage of blood neutrophils in a similar mouse model of allergic asthma, implying a possible role for CP as a biomarker in asthma [99]. 

An interesting study evaluated the association of early-age fecal CP levels with the later development of allergic diseases in children from farming and non-farming environments. Additionally, the effect of the gut microbiota on fecal CP levels was investigated [100]. The presence of CP in feces was used to detect intestinal inflammation. It was observed that a high level of intestinal inflammation at two months of age predicted asthma and atopic dermatitis (AD) at six years of age, and was associated with a low abundance of fecal *E. coli*. Reduced fecal *E. coli* colonization and impaired IL-10 activation may account for the intestinal inflammation associated with high fecal CP, and the subsequent risk of asthma and AD. These studies highlight the potential role of CP in predicting subsequent asthma and suggest that CP has a variable effect on asthmatic airways depending on the inflammatory endotype.

### 7.4. CP in Other Lung Diseases

Patients with systemic sclerosis (SSc) and dysbiosis have higher fecal CP levels and are more frequently affected by interstitial lung disease (ILD), implying that fecal CP serves as a marker of an abnormal immune response and increases the risk of ILD [101]. Volkman hypothesized that altering the gut microbiota of SSc patients perpetuates inflammation and fibrosis via a possible lung–gut crosstalk mechanism or the production of molecules that travel through the lung to mediate damage [102]. During infections, high CP levels were also found in the lungs, in addition to high fecal CP [103]. 

### 7.5. CP in Children with Obstructive Sleep Apnoea (OSA)

Obstructive sleep apnoea (OSA) is a condition defined by repeated episodes of partial or complete obstruction of the upper airway during sleep, resulting in disruption of normal ventilation, hypoxemia, and sleep fragmentation [104]. Pediatric OSA has been identified as a condition associated with an increased risk of cardiovascular morbidity and metabolic dysfunction, particularly in obese children [105]. The recurrent hypoxia re-oxygenation events and sleep fragmentation observed in children with OSAs are thought to increase the generation of reactive oxygen species (ROS) and systemic inflammation, which are thought to play a role in the acceleration and propagation of atherogenesis [106].

As a corollary to these speculations, Kim et al. focused on CP and found that children with OSA have elevated morning plasma CP levels. These levels exhibit dependencies on the severity of OSAs, even in children who are not obese. Furthermore, CP levels were found to be highly correlated not only with CRP and IL-6, but also with endothelial function [107]. Additional research is needed to confirm these findings and to investigate the intrinsic value of assessing CP levels in the context of evaluating children at risk for OSA.

## 8. Conclusions 

The pediatric respiratory community has been in search of new, affordable, easy-to-use, and accountable biomarkers for many years. The difficulties in defining exacerbation, predicting future exacerbations, and halting the overuse of antibiotics in many pediatric respiratory diseases have made the need more imperative. CP is a potential biomarker that could be implemented in medical practice with the aim of giving answers where there were not one before. Research on CP in blood, sputum or BAL in respiratory diseases in children is mandatory to better clarify the role of CP in pediatric respiratory medicine.

## Figures and Tables

**Table 1:** Current concepts of calprotectin release.

Cell Type	Mode of Release
Monocyte	Actively: Independent of Golgi pathway; Endolysosomes and secretory lysosomes are involved. Passively: Necrosis.
Neutrophil	Actively: Independent of Golgi pathway; Passively: Necrosis; NET formation.

NET, neutrophil extracellular traps.

**Table 2:** CP in infectious diseases, human studies.

Authors	Age of Cohort	Study Results
Fang, P. et al. [79]	adults	S100A8 heterodimer is helpful in community-acquired pneumonia diagnosis and severity assessment
Siljan, W.W. et al. [81]	adults	CP identifies bacterial vs. viral community acquired pneumonia and predicts the outcome
Wu, K.A. et al. [86]	adults	CP contributes in noninvasive diagnosis of complicated parapneumonic effusions and empyema
Mohammed, O.M. et al. [87]	adults	CP helps in differentiation between benign and malignant pleural effusion
Xu, D. et al. [88]	adults	S100A9 heterodimer contributes to early TB diagnosis
Liu, Q. et al. [89]	adults	CP value correlates with TB development
Jerkic, S.P. et al. [90]	children and young adults (6.2 to 27.3 years of age)	CP reflects ongoing neutrophilic inflammation in patients with obliterative bronchiolitis
Havelka, A. et al. [78]	adults	CP is a better discriminator between bacterial and viral infections compared to HBP and PCT

**Table 3:** CP in infectious diseases, animal studies.

Authors	Species	Study Results
Achuiti et al. [80]	mice	CP deficiency aggravates *staphylococcal* pneumonia
Achuiti et al. [55]	mice	CP protects against sepsis secondary to Gram-negative bacterial pneumonia

## Abbreviations

Calprotectin (CP), thymic stromal lymphopoietin (TSLP), damage-associated molecular patterns (DAMPs), acute respiratory infections (ARIs), procalcitonin (PCT), heparin-binding protein (HBP), community-acquired pneumonia (CAP), paraneumonic effusion (PPE), bronchiolitis obliterans (BO), cystic fibrosis (CF), obstructive sleep apnoea (OSA), interstitial lung disease (ILD).

## References

[1] Moore B.W. (1965). A soluble protein characteristic of the nervous system. Biochem. Biophys. Res. Commun..

[2] Vogl T., Gharibyan A.L., Morozova-Roche L.A. (2012). Pro-inflammatory S100A8 and S100A9 proteins: Self-assembly into multifunctional native and amyloid complexes. Int. J. Mol. Sci..

[3] Rammes A., Roth J., Goebeler M., Klempt M., Hartmann M., Sorg C. (1997). Myeloid-related protein (MRP) 8 and MRP14, calcium-binding proteins of the S100 family, are secreted by activated monocytes via a novel, tubulin-dependent pathway. J. Biol. Chem..

[4] Pruenster M., Vogl T., Roth J., Sperandio M. (2016). S100A8/A9: From basic science to clinical application. Pharmacol. Ther..

[5] Sander J., Fagerhol M.K., Bakken J.S., Dale I. (1984). Plasma levels of the leucocyte L1 protein in febrile conditions: Relation to aetiology, number of leucocytes in blood, blood sedimentation reaction and C-reactive protein. Scand. J. Clin. Lab. Investig..

[6] Marenholz I., Lovering R.C., Heizmann C.W. (2006). An update of the S100 nomenclature. Biochim. Biophys. Acta.

[7] Loomans H.J., Hahn B.L., Li Q.Q., Phadnis S.H., Sohnle P.G. (1998). Histidine-based zinc-binding sequences and the antimicrobial activity of calprotectin. J. Infect. Dis..

[8] Roth J., Burwinkel F., van den Bos C., Goebeler M., Vollmer E., Sorg C. (1993). MRP8 and MRP14, S-100-like proteins associated with myeloid differentiation, are translocated to plasma membrane and intermediate filaments in a calcium-dependent manner. Blood.

[9] Fritz G., Botelho H.M., Morozova-Roche L.A., Gomes C.M. (2010). Natural and amyloid self-assembly of S100 proteins: Structural basis of functional diversity. FEBS J..

[10] Damo S.M., Kehl-Fie T.E., Sugitani N., Holt M.E., Rathi S., Murphy W.J., Zhang Y., Betz C., Hench L., Fritz G. (2013). Molecular basis for manganese sequestration by calprotectin and roles in the innate immune response to invading bacterial pathogens. Proc. Natl. Acad. Sci. USA.

[11] Corbin B.D., Seeley E.H., Raab A., Feldmann J., Miller M.R., Torres V.J., Anderson K.L., Dattilo B.M., Dunman P.M., Gerads R. (2008). Metal chelation and inhibition of bacterial growth in tissue abscesses. Science.

[12] Vogl T., Leukert N., Barczyk K., Strupat K., Roth J. (2006). Biophysical characterization of S100A8 and S100A9 in the absence and presence of bivalent cations. Biochim. Biophys. Acta.

[13] Leukert N., Vogl T., Strupat K., Reichelt R., Sorg C., Roth J. (2006). Calcium-dependent tetramer formation of S100A8 and S100A9 is essential for biological activity. J. Mol. Biol..

[14] Vogl T., Tenbrock K., Ludwig S., Leukert N., Ehrhardt C., van Zoelen M.A., Nacken W., Foell D., van der Poll T., Sorg C. (2007). Mrp8 and Mrp14 are endogenous activators of Toll-like receptor 4, promoting lethal, endotoxin-induced shock. Nat. Med..

[15] Schäfer B.W., Wicki R., Engelkamp D., Mattei M.G., Heizmann C.W. (1995). Isolation of a YAC clone covering a cluster of nine S100 genes on human chromosome 1q21: Rationale for a new nomenclature of the S100 calcium-binding protein family. Genomics.

[16] Voganatsi A., Panyutich A., Miyasaki K.T., Murthy R.K. (2001). Mechanism of extracellular release of human neutrophil calprotectin complex. J. Leukoc. Biol..

[17] Urban C.F., Ermert D., Schmid M., Abu-Abed U., Goosmann C., Nacken W., Brinkmann V., Jungblut P.R., Zychlinsky A. (2009). Neutrophil extracellular traps contain calprotectin, a cytosolic protein complex involved in host defense against Candida albicans. PLoS Pathog..

[18] Lemarchand P., Vaglio M., Mauël J., Markert M. (1992). Translocation of a small cytosolic calcium-binding protein (MRP-8) to plasma membrane correlates with human neutrophil activation. J. Biol. Chem..

[19] Hetland G., Talgö G.J., Fagerhol M.K. (1998). Chemotaxins C5a and fMLP induce release of calprotectin (leucocyte L1 protein) from polymorphonuclear cells in vitro. Mol. Pathol..

[20] Frosch M., Metze D., Foell D., Vogl T., Sorg C., Sunderkötter C., Roth J. (2005). Early activation of cutaneous vessels and epithelial cells is characteristic of acute systemic onset juvenile idiopathic arthritis. Exp. Dermatol..

[21] Ley K., Laudanna C., Cybulsky M.I., Nourshargh S. (2007). Getting to the site of inflammation: The leukocyte adhesion cascade updated. Nat. Rev. Immunol..

[22] Schmidt S., Moser M., Sperandio M. (2013). The molecular basis of leukocyte recruitment and its deficiencies. Mol. Immunol..

[23] Pruenster M., Kurz A.R., Chung K.J., Cao-Ehlker X., Bieber S., Nussbaum C.F., Bierschenk S., Eggersmann T.K., Rohwedder I., Heinig K. (2015). Extracellular MRP8/14 is a regulator of β2 integrin-dependent neutrophil slow rolling and adhesion. Nat. Commun..

[24] Zwadlo G., Schlegel R., Sorg C. (1986). A monoclonal antibody to a subset of human monocytes found only in the peripheral blood and inflammatory tissues. J. Immunol..

[25] Hessian P.A., Edgeworth J., Hogg N. (1993). MRP-8 and MRP-14, two abundant Ca (2+)-binding proteins of neutrophils and monocytes. J. Leukoc. Biol..

[26] Brandtzaeg P., Dale I., Fagerhol M.K. (1987). Distribution of a formalin-resistant myelomonocytic antigen (L1) in human tissues. I. Comparison with other leukocyte markers by paired immunofluorescence and immunoenzyme staining. Am. J. Clin. Pathol..

[27] Shabani F., Farasat A., Mahdavi M., Gheibi N. (2018). Calprotectin (S100A8/S100A9): A key protein between inflammation and cancer. Inflamm. Res..

[28] Nilsen T., Sundström J., Lind L., Larsson A. (2014). Serum calprotectin levels in elderly males and females without bacterial or viral infections. Clin. Biochem..

[29] Calcaterra V., De Amici M., Leonard M.M., De Silvestri A., Pelizzo G., Buttari N., Michev A., Leggio M., Larizza D., Cena H. (2018). Serum Calprotectin Level in Children: Marker of Obesity and its Metabolic Complications. Ann. Nutr. Metab..

[30] Pillay S.N., Asplin J.R., Coe F.L. (1998). Evidence that calgranulin is produced by kidney cells and is an inhibitor of calcium oxalate crystallization. Am. J. Physiol..

[31] Garg M., Leach S.T., Coffey M.J., Katz T., Strachan R., Pang T., Needham B., Lui K., Ali F., Day A.S. (2017). Age-dependent variation of fecal calprotectin in cystic fibrosis and healthy children. J. Cyst. Fibros..

[32] Zhu Q., Li F., Wang J., Shen L., Sheng X. (2016). Fecal Calprotectin in Healthy Children Aged 1-4 Years. PLoS ONE.

[33] Joshi S., Lewis S.J., Creanor S., Ayling R.M. (2010). Age-related faecal calprotectin, lactoferrin and tumour M2-PK concentrations in healthy volunteers. Ann. Clin. Biochem..

[34] Medzhitov R. (2008). Origin and physiological roles of inflammation. Nature.

[35] Medeiros G.F., Mendes A., Castro R.A., Baú E.C., Nader H.B., Dietrich C.P. (2000). Distribution of sulfated glycosaminoglycans in the animal kingdom: Widespread occurrence of heparin-like compounds in invertebrates. Biochim. Biophys. Acta.

[36] Gallagher J.T., Lyon M., Iozzo R.V. (2000). Molecular structure of heparan sulfate and interactions with growth factors and morphogens. Proteoglycans: Structure, Biology and Molecular Interactions.

[37] Buzza M.S., Zamurs L., Sun J., Bird C.H., Smith A.I., Trapani J.A., Froelich C.J., Nice E.C., Bird P.I. (2005). Extracellular matrix remodeling by human granzyme B via cleavage of vitronectin, fibronectin, and laminin. J. Biol. Chem..

[38] Hallak L.K., Spillmann D., Collins P.L., Peeples M.E. (2000). Glycosaminoglycan sulfation requirements for respiratory syncytial virus infection. J. Virol..

[39] Clausen T.M., Sandoval D.R., Spliid C.B., Pihl J., Perrett H.R., Painter C.D., Narayanan A., Majowicz S.A., Kwong E.M., McVicar R.N. (2020). SARS-CoV-2 Infection Depends on Cellular Heparan Sulfate and ACE2. Cell.

[40] Robinson M.J., Tessier P., Poulsom R., Hogg N. (2002). The S100 family heterodimer, MRP-8/14, binds with high affinity to heparin and heparan sulfate glycosaminoglycans on endothelial cells. J. Biol. Chem..

[41] Eue I., Sorg C. (2001). Arachidonic acid specifically regulates binding of S100A8/9, a heterodimer complex of the S100 class of calcium binding proteins, to human microvascular endothelial cells. Atherosclerosis.

[42] Newton R.A., Hogg N. (1998). The human S100 protein MRP-14 is a novel activator of the beta 2 integrin Mac-1 on neutrophils. J. Immunol..

[43] Saenz S.A., Taylor B.C., Artis D. (2008). Welcome to the neighborhood: Epithelial cell-derived cytokines license innate and adaptive immune responses at mucosal sites. Immunol. Rev..

[44] Kouzaki H., O’Grady S.M., Lawrence C.B., Kita H. (2009). Proteases induce production of thymic stromal lymphopoietin by airway epithelial cells through protease-activated receptor-2. J. Immunol..

[45] Wang Y.H., Angkasekwinai P., Lu N., Voo K.S., Arima K., Hanabuchi S., Hippe A., Corrigan C.J., Dong C., Homey B. (2007). IL-25 augments type 2 immune responses by enhancing the expansion and functions of TSLP-DC-activated Th2 memory cells. J. Exp. Med..

[46] Kato T., Kouzaki H., Matsumoto K., Hosoi J., Shimizu T. (2017). The effect of calprotectin on TSLP and IL-25 production from airway epithelial cells. Allergol. Int..

[47] Chan J.K., Roth J., Oppenheim J.J., Tracey K.J., Vogl T., Feldmann M., Horwood N., Nanchahal J. (2012). Alarmins: Awaiting a clinical response. J. Clin. Investig..

[48] Watanabe T., Asai K., Fujimoto H., Tanaka H., Kanazawa H., Hirata K. (2011). Increased levels of HMGB-1 and endogenous secretory RAGE in induced sputum from asthmatic patients. Respir. Med..

[49] Ullah M.A., Loh Z., Gan W.J., Zhang V., Yang H., Li J.H., Yamamoto Y., Schmidt A.M., Armour C.L., Hughes J.M. (2014). Receptor for advanced glycation end products and its ligand high-mobility group box-1 mediate allergic airway sensitization and airway inflammation. J. Allergy Clin. Immunol..

[50] Idzko M., Hammad H., van Nimwegen M., Kool M., Willart M.A., Muskens F., Hoogsteden H.C., Luttmann W., Ferrari D., Di Virgilio F. (2007). Extracellular ATP triggers and maintains asthmatic airway inflammation by activating dendritic cells. Nat. Med..

[51] Wang S., Song R., Wang Z., Jing Z., Wang S., Ma J. (2018). S100A8/A9 in Inflammation. Front Immunol..

[52] Kehl-Fie T.E., Skaar E.P. (2010). Nutritional immunity beyond iron: A role for manganese and zinc. Curr. Opin. Chem. Biol..

[53] Clark H.L., Jhingran A., Sun Y., Vareechon C., de Jesus Carrion S., Skaar E.P., Chazin W.J., Calera J.A., Hohl T.M., Pearlman E. (2016). Zinc and Manganese Chelation by Neutrophil S100A8/A9 (Calprotectin) Limits Extracellular Aspergillus Fumigatus Hyphal Growth and Corneal Infection. J. Immunol..

[54] Simard J.C., Simon M.M., Tessier P.A., Girard D. (2011). Damage-associated molecular pattern S100A9 increases bactericidal activity of human neutrophils by enhancing phagocytosis. J. Immunol..

[55] Achouiti A., Vogl T., Urban C.F., Röhm M., Hommes T.J., van Zoelen M.A., Florquin S., Roth J., van ‘t Veer C., de Vos A.F. (2012). Myeloid-related protein-14 contributes to protective immunity in gram-negative pneumonia derived sepsis. PLoS Pathog..

[56] Tsai S.Y., Segovia J.A., Chang T.H., Morris I.R., Berton M.T., Tessier P.A., Tardif M.R., Cesaro A., Bose S. (2014). DAMP molecule S100A9 acts as a molecular pattern to enhance inflammation during influenza a virus infection: Role of DDX21-TRIF-TLR4-MyD88 pathway. PLoS Pathog..

[57] De Jong H.K., Achouiti A., Koh G.C., Parry C.M., Baker S., Faiz M.A., van Dissel J.T., Vollaard A.M., van Leeuwen E.M.M., Roelofs J.J.T.H. (2015). Expression and function of S100A8/A9 (calprotectin) in human typhoid fever and the murine Salmonella model. PLoS Negl. Trop Dis..

[58] Raju M.S., Kamaraju R.S., Sritharan V., Rajkumar K., Natarajan S., Kumar A.D., Burgula S. (2016). Continuous evaluation of changes in the serum proteome from early to late stages of sepsis caused by Klebsiella pneumoniae. Mol. Med. Rep..

[59] Ometto F., Friso L., Astorri D., Botsios C., Raffeiner B., Punzi L., Doria A. (2017). Calprotectin in rheumatic diseases. Exp. Biol. Med..

[60] Coveney A.P., Wang W., Kelly J., Liu J.H., Blankson S., Wu Q.D., Redmond H.P., Wang J.H. (2015). Myeloid-related protein 8 induces self-tolerance and cross-tolerance to bacterial infection via TLR4- and TLR2-mediated signal pathways. Sci. Rep..

[61] Lorey M.B., Rossi K., Eklund K.K., Nyman T.A., Matikainen S. (2017). Global Characterization of Protein Secretion from Human Macrophages Following Non-canonical Caspase-4/5 Inflammasome Activation. Mol. Cell Proteom..

[62] Pouwels S.D., Nawijn M.C., Bathoorn E., Riezebos-Brilman A., van Oosterhout A.J., Kerstjens H.A., Heijink I.H. (2015). Increased serum levels of LL37, HMGB1 and S100A9 during exacerbation in COPD patients. Eur. Respir. J..

[63] Walker C.L.F., Rudan I., Liu L., Nair H., Theodoratou E., Bhutta Z.A., O’Brien K.L., Campbell H., Black R.E. (2013). Global burden of childhood pneumonia and diarrhoea. Lancet.

[64] Kassebaum N., Kyu H.H., Zoeckler L., Olsen H.E., Thomas K., Pinho C., Global Burden of Disease Child and Adolescent Health Collaboration (2017). Child and Adolescent Health From 1990 to 2015: Findings From the Global Burden of Diseases, Injuries, and Risk Factors 2015 Study. JAMA Pediatr..

[65] United Nations Children’s Fund UNICEF; New York, “The State of the World’s Children 2016: A Fair Chance for Every Child”, 2016. https://www.unicef.org/publications/files/UNICEF_SOWC_2016.pdf.

[66] Nair H., Simões E.A., Rudan I., Gessner B.D., Azziz-Baumgartner E., Zhang J.S.F., Feikin D.R., Mackenzie G.A., Moiisi J.C., Roca A. (2013). Severe Acute Lower Respiratory Infections Working Group. Global and regional burden of hospital admissions for severe acute lower respiratory infections in young children in 2010: A systematic analysis. Lancet.

[67] GBD 2015 LRI Collaborators (2017). Estimates of the global, regional, and national morbidity, mortality, and aetiologies of lower respiratory tract infections in 195 countries: A systematic analysis for the Global Burden of Disease Study 2015. Lancet Infect Dis..

[68] Histoshi T., McAllister D.A., O’Brien K.L., Simoes E.A.F., Madhi S.A., Gessner B.D., Polack F.P., Balsells E., Acacio S., Aguayo C. (2017). Global, regional, and national disease burden estimates of acute lower respiratory infections due to respiratory syncytial virus in young children in 2015: A systematic review and modelling study. Lancet.

[69] Dupuy A.-M., Philippart F., Péan Y., Lasocki S., Charles P.-E., Chalumeau M., Claessens Y.-E., Quenot J.-P., Guen C.G.-L., Ruiz S. (2013). Role of biomarkers in the management of antibiotic therapy: An expert panel review: I—Currently available biomarkers for clinical use in acute infections. Ann. Intensive Care.

[70] Spellberg B., Gilbert D.N. (2014). The future of antibiotics and resistance: A tribute to a career of leadership by John Bartlett. Clin. Infect Dis..

[71] Morley D., Torres A., Cillóniz C., Martin-Loeches I. (2017). Predictors of treatment failure and clinical stability in patients with community acquired pneumonia. Ann. Transl. Med..

[72] Biomarkers Definitions Working Group (2001). Biomarkers and surrogate endpoints: Preferred definitions and conceptual framework. Clin. Pharmacol. Ther..

[73] Karakioulaki M., Stolz D. (2019). Biomarkers in Pneumonia-Beyond Procalcitonin. Int. J. Mol. Sci..

[74] Shaddock E.J. (2016). How and when to use common biomarkers in community-acquired pneumonia. Pneumonia.

[75] Heiskanen-Kosma T., Korppi M. (2000). Serum C-reactive protein cannot differentiate bacterial and viral aetiology of community-acquired pneumonia in children in primary healthcare settings. Scand. J. Infect Dis..

[76] Ito A., Ishida T. (2020). Diagnostic markers for community-acquired pneumonia. Ann. Transl. Med..

[77] Lipcsey M., Hanslin K., Stålberg J., Smekal D., Larsson A. (2019). The time course of calprotectin liberation from human neutrophil granulocytes after *Escherichia coli* and endotoxin challenge. Innate Immun..

[78] Havelka A., Sejersen K., Venge P., Pauksens K., Larsson A. (2020). Calprotectin, a new biomarker for diagnosis of acute respiratory infections. Sci. Rep..

[79] Fang P., Zheng L., Cao P., Zhang C., Fei J., Xu Z., Feng C.-M., Zhao H., Lu Y.-J., Fu L. (2021). Serum S100A8 as an early diagnostic biomarker in patients with community-acquired pneumonia. Arch. Med. Sci..

[80] Achouiti A., Vogl T., Van der Meer A.J., Stroo I., Florquin S., de Boer O.J., Roth J., Zeerleder S., van ‘t Veer C., de Vos A.F. (2015). Myeloid-related protein-14 deficiency promotes inflammation in staphylococcal pneumonia. Eur. Respir. J..

[81] Siljan W.W., Holter J.C., Michelsen A.E., Nymo S.H., Lauritzen T., Oppen K., Husebye E., Ueland T., Mollnes T.E., Aukrust P. (2019). Inflammatory biomarkers are associated with aetiology and predict outcomes in community-acquired pneumonia: Results of a 5-year follow-up cohort study. ERJ Open Res..

[82] McCauley L., Dean N. (2015). Pneumonia and empyema: Causal, casual or unknown. J. Thorac. Dis..

[83] Corcoran J.P., Wrightson J.M., Belcher E., DeCamp M.M., Feller-Kopman D., Rahman N.M. (2015). Pleural infection: Past, present, and future directions. Lancet Respir. Med..

[84] Light R.W. (2006). Parapneumonic effusions and empyema. Proc. Am. Thorac. Soc..

[85] Hampson C., Lemos J.A., Klein J.S. (2008). Diagnosis and management of parapneumonic effusions. Semin. Respir. Crit. Care Med..

[86] Wu K.A., Wu C.C., Liu Y.C., Hsueh P.C., Chin C.Y., Wang C.L., Chu C.M., Shih L.J., Yang C.Y. (2019). Combined serum biomarkers in the noninvasive diagnosis of complicated parapneumonic effusions and empyema. BMC Pulm. Med..

[87] Mohammed O.M., Hussein K.M., Ramadan A.E., Mahmoud G.T., El-Naggar M.E., Gaber N.E.Z. (2019). Diagnostic value of calprotectin in differentiation between benign and malignant pleural effusion. Egypt. J. Bronchol..

[88] Xu D., Li Y., Li X., Wei L.L., Pan Z., Jiang T.T., Chen Z.L., Wang C., Cao W.M., Zhang X. (2015). Serum protein S100A9, SOD3, and MMP9 as new diagnostic biomarkers for pulmonary tuberculosis by iTRAQ-coupled two-dimensional LC-MS/MS. Proteomics.

[89] Liu Q., Pan L., Han F., Luo B., Jia H., Xing A., Li Q., Zhang Z. (2018). Proteomic profiling for plasma biomarkers of tuberculosis progression. Mol. Med. Rep..

[90] Jerkic S.P., Michel F., Donath H., Herrmann E., Schubert R., Rosewich M., Zielen S. (2020). Calprotectin as a New Sensitive Marker of Neutrophilic Inflammation in Patients with Bronchiolitis Obliterans. Mediat. Inflamm..

[91] Wilson G.B., Fudenberg H.H., Jahn T.L. (1975). Studies on cystic fibrosis using isoelectric focusing. I. An assay for detection of cystic fibrosis homozygotes and heterozygote carriers from serum. Pediatr. Res..

[92] Dorin J.R., Novak M., Hill R.E., Brock D.J., Secher D.S., van Heyningen V. (1987). A clue to the basic defect in cystic fibrosis from cloning the CF antigen gene. Nature.

[93] Gray R.D., Imrie M., Boyd A.C., Porteous D., Innes J.A., Greening A.P. (2010). Sputum and serum calprotectin are useful biomarkers during CF exacerbation. J. Cyst. Fibros..

[94] Schnapp Z., Hartman C., Livnat G., Shteinberg M., Elenberg Y. (2019). Decreased Fecal Calprotectin Levels in Cystic Fibrosis Patients After Antibiotic Treatment for Respiratory Exacerbation. J. Pediatr. Gastroenterol. Nutr..

[95] Reid P.A., McAllister D.A., Boyd A.C., Innes J.A., Porteous D., Greening A.P., Gray R.D. (2015). Measurement of serum calprotectin in stable patients predicts exacerbation and lung function decline in cystic fibrosis. Am. J. Respir. Crit. Care Med..

[96] Gray R.D., MacGregor G., Noble D., Imrie M., Dewar M., Boyd A.C., Innes J.A., Porteous D.J., Greening A.P. (2008). Sputum proteomics in inflammatory and suppurative respiratory diseases. Am. J. Respir. Crit. Care Med..

[97] Lee T.H., Jang A.S., Park J.S., Kim T.H., Choi Y.S., Shin H.R., Park S.W., Uh S.T., Choi J.S., Kim Y.H. (2013). Elevation of S100 calcium binding protein A9 in sputum of neutrophilic inflammation in severe uncontrolled asthma. Ann. Allergy Asthma. Immunol..

[98] Palmer L.D., Maloney K.N., Boyd K.L., Goleniewska A.K., Toki S., Maxwell C.N., Chazin W.J., Peebles R.S., Newcomb D.C., Skaar E.P. (2019). The Innate Immune Protein S100A9 Protects from T-Helper Cell Type 2-mediated Allergic Airway Inflammation. Am. J. Respir. Cell Mol. Biol..

[99] Lee Y.G., Hong J., Lee P.H., Lee J., Park S.W., Kim D., Jang A.S. (2020). Serum Calprotectin Is a Potential Marker in Patients with Asthma. J. Korean Med. Sci..

[100] Orivuori L., Mustonen K., de Goffau M.C., Hakala S., Paasela M., Roduit C., Dalphin J.C., Genuneit J., Lauener R., Riedler J. (2015). High level of fecal calprotectin at age 2 months as a marker of intestinal inflammation predicts atopic dermatitis and asthma by age 6. Clin. Exp. Allergy.

[101] Andréasson K., Alrawi Z., Persson A., Jönsson G., Marsal J. (2016). Intestinal dysbiosis is common in systemic sclerosis and associated with gastrointestinal and extraintestinal features of disease. Arthritis Res. Ther..

[102] Volkmann E.R. (2017). Intestinal microbiome in scleroderma: Recent progress. Curr. Opin. Rheumatol..

[103] Raquil M.A., Anceriz N., Rouleau P., Tessier P.A. (2008). Blockade of antimicrobial proteins S100A8 and S100A9 inhibits phagocyte migration to the alveoli in streptococcal pneumonia. J. Immunol..

[104] Kushida C.A., Chediak A., Berry R.B., Brown L.K., Gozal D., Iber C., Parthasarathy S., Quan S.F., Rowley J.A., Positive Airway Pressure Titration Task Force, American Academy of Sleep Medicine (2008). Clinical guidelines for the manual titration of positive airway pressure in patients with obstructive sleep apnea. J. Clin. Sleep Med..

[105] Bhattacharjee R., Kheirandish-Gozal L., Pillar G., Gozal D. (2009). Cardiovascular complications of obstructive sleep apnea syndrome: Evidence from children. Prog. Cardiovasc. Dis..

[106] Atkeson A., Yeh S.Y., Malhotra A., Jelic S. (2009). Endothelial function in obstructive sleep apnea. Prog. Cardiovasc. Dis..

[107] Kim J., Bhattacharjee R., Snow A.B., Capdevila O.S., Kheirandish-Gozal L., Gozal D. (2010). Myeloid-related protein 8/14 levels in children with obstructive sleep apnoea. Eur. Respir. J..

